# Comparative Effects of Microalgal Incorporation on the Rheological, Microstructural, and Colorimetric Behavior of Potato Starch Gels

**DOI:** 10.3390/foods15162932

**Published:** 2026-08-21

**Authors:** Sally Fawaz, Francesc Sepulcre, Amira Haddarah, Abderahman Rejeb

**Affiliations:** 1 Departament d’Enginyeria Agroalimentària i Biotecnologia, Universitat Politècnica de Catalunya, Campus del Baix Llobregat, Carrer Esteve Terradas 8, Castelldefels, 08860 Barcelona, Spain; francesc.sepulcre@upc.edu; 2 Doctoral School of Sciences and Technology, Lebanese University, Rafic Hariri Campus, Hadath 1683, Lebanon; amirahaddara@hotmail.com; 3 Kautz Gyula Faculty of Business and Economics, Széchenyi István University, 9026 Győr, Hungary

**Keywords:** *Arthrospira platensis*, *Chlorella vulgaris*, colorimetry, gel microstructure, thixotropy, viscosity

## Abstract

The development of sustainable, nutrient-dense food systems requires a comprehensive understanding of how microalgae influence the mechanical properties of starch hydrogels. While certain microalgae are common food additives, a critical research gap remains regarding their effect on the rheological behavior and structural integrity of potato starch gels specifically. This study addressed this gap by evaluating the mechanical, microstructural and optical impacts of *Arthrospira platensis* (commonly known as Spirulina) and *Chlorella vulgaris* at 0.5%, 1% and 2% (*w*/*w*). Utilizing steady-shear flow tests, colorimetry, NIR spectroscopy and microscopy, we characterized changes in steady rheological parameters, color, chemical changes and microstructure of fortified hydrogels. Results indicated that filamentous *Arthrospira platensis* reinforces the matrix, significantly increasing yield stress from 10.2 Pa in the control to 32.4 Pa at 2% inclusion. In contrast, spherical *Chlorella vulgaris* appears to act as a structural filler, reducing yield stress to 4.8 Pa at 2%. Microscopy confirmed these morphological influences, showing *Arthrospira platensis* filaments entangling granules while *Chlorella* cells integrated into inter-granular spaces. Colorimetry revealed significant darkening (L* decreased from 31.59 to 18.54 at 2% *Spirulina* addition) and significant greening (*p* < 0.05). NIR spectroscopy demonstrated potential physical interactions via vibrational markers at 5172 cm^−1^ and 5646 cm^−1^, indicating water matrix redistribution within the system. This research demonstrates how incorporating *Spirulina* and *Chlorella vulgaris* provides a viable approach for modifying the physical properties of starch-based matrices. The findings indicate that *Spirulina* enhances flow resistance and structural stability under steady shear, whereas *Chlorella vulgaris* reduces flow barriers, thereby increasing the spreadability of these composite food systems.

## 1. Introduction

Starch gels play a central role in determining the structure, stability and sensory attributes of many food products. Leveraging its inherent functional characteristics, the food sector utilizes starch extensively in the formulation of gelled structures and as a stabilizing thickener [1,2]. Furthermore, it serves as a carrier agent during the spray-drying process for concentrated fruit extracts and in the production of commercial starch syrups [3,4,5].

Potato starch, in particular, is valued for its high swelling capacity, phosphate-mediated hydration and ability to form cohesive, translucent networks, making it a suitable model for studying how compositional changes influence gelatinization and gel development. In addition, it exhibits superior stickiness and exceptional paste clarity compared to other botanical starch varieties. These distinctive functional attributes facilitate its broad application throughout the food sector [5,6,7].

In parallel, the growing interest in nutrient-dense, minimally processed ingredients has brought microalgae to the forefront as natural sources of proteins, pigments, fibers and bioactive compounds. Species such as *Arthrospira platensis* and *Chlorella vulgaris* are already incorporated into beverages, bakery products, snacks and emulsions, reflecting their versatility and functional potential in food systems [8]. Moreover, their interactions with different starch matrices during gelatinization are still not fully understood.

Microalgal biomass contains a combination of proteins, dietary fibers, polyunsaturated fatty acids and pigments that can interact with starch granules or modify water distribution during heating. These components influence key starch processes like granule swelling, amylose leaching, and network formation. Several studies have shown that microalgae can alter the rheology and microstructure of mixed biopolymer systems. For example, microalgal addition has been reported to modify viscoelasticity and network formation in starch–carrageenan gels and to influence the gelation behavior of composite matrices [9,10].

The contrasting macro-compositional and anatomical profiles of *Arthrospira platensis* and *Chlorella vulgaris* suggest that their integration into a highly swelling hydrocolloid matrix will trigger distinctly different structural consequences. *Arthrospira platensis* is characterized by an exceptionally high protein content (~70%), predominantly composed of highly water-soluble phycobiliproteins, alongside a fragile, multi-layered cell wall made of peptidoglycan. In contrast, *Chlorella vulgaris* exhibits a lower protein fraction (~60.00%) coupled with substantially higher lipid content (~12.00%) and a rigid, multi-layered cell wall composed of an insoluble cellulose–glucosamine matrix and sporopollenin. These variations alter how each biomass interacts with available moisture; the abundant hydrophilic domains of soluble *Arthrospira platensis* proteins promote rapid water absorption, whereas the robust cell walls and lipid fractions of *Chlorella vulgaris* present a more hydrophobic, intact particulate barrier within the aqueous phase [9,11].

When blended with potato starch, these species-specific components are hypothesized to physically interact with the mechanics of starch gelatinization and subsequent gel network formation through a multi-faceted physical composite effect. The massive influx of hydrophilic microalgal proteins, together with complex structural carbohydrates and dietary fibers (such as cellulose, hemicellulose and pectin), initiates a state of competitive hydration that effectively strips bulk water away from the native starch granules. This moisture restriction limits the thermodynamic swelling capacity of potato starch, which is inherently sensitive to water availability due to its phosphate-mediated hydration pathways. Simultaneously, the structural wall polysaccharides remain largely intact during thermal processing, possibly acting as rigid, non-cohesive filler particles that physically disrupt the continuity of the continuous amylose–amylopectin network. Concurrently, soluble proteins tend to undergo thermal unfolding and phase-separate into distinct micro-domains within interstitial spaces, while liberated lipids can form inclusive amylose composites with leached linear amylose chains, structurally restricting amylose retrogradation and potentially altering the long-term stability and viscoelastic development of composite hydrogels.

Work on isolated protein fractions from *Arthrospira platensis* and *Chlorella vulgaris* has further highlighted their emulsifying, water-binding and foaming properties, suggesting that even low levels of biomass may affect starch gel development [12].

Beyond these systems, microalgae have also been incorporated into starch-based matrices of different botanical origins. A recent study using rice starch reported that microalgal biomass altered pasting behavior, gel firmness and color attributes, indicating that microalgae can interfere with starch gelatinization and network formation even at low inclusion levels. These effects appear to depend strongly on the physicochemical characteristics of the starch, as rice starch- low in phosphate and with limited swelling-responds differently from tuber starches [13]. Potato starch, with its pronounced swelling power and sensitivity to compositional perturbations, is therefore likely to exhibit distinct interactions with microalgal components, yet this has not been systematically investigated.

Microalgae have also been explored as structuring agents in cereal-based and printed foods. Incorporation of *Arthrospira platensis* into gluten-free pasta has been shown to modify dough rheology and enhance bioactive compound content without compromising texture [11]. In 3D-printed cookie doughs, the addition of *Arthrospira platensis* and *Chlorella vulgaris* improved mechanical resistance, elasticity and printability, demonstrating their potential to influence both processing behavior and final structure [14]. Collectively. These findings suggest that microalgae can affect water mobility, polymer interactions and network formation—key aspects of hydrocolloid functionality.

Despite this growing body of work, the specific influence of whole microalgal biomass on potato starch gels remains largely unexplored. Most available studies focus on cereal starches, protein–polysaccharide systems or isolated protein fractions rather than on whole biomass interacting with a highly swelling, phosphate-rich starch such as potato starch. Moreover, *Arthrospira platensis* and *Chlorella vulgaris* differ substantially in protein content, pigment composition and cell-wall structure, suggesting that their effects on gelatinization and gelation may not be equivalent. Understanding these interactions is essential for determining whether whole microalgae act solely as nutrient carriers or whether they also impart physical property modifications capable of altering water distribution, granule integrity and viscoelastic development. This knowledge is particularly relevant for clean-label formulation, plant-based innovation and the design of sustainable texturizing strategies.

In this study, potato starch gels were fortified with different concentrations of *Arthrospira platensis* and *Chlorella vulgaris* to evaluate their impact on gelatinization and final gel properties. The choice to utilize the whole biomass—rather than isolated fractions—was a deliberate strategic point central to this study’s design. From a sustainability and economic standpoint, utilizing the intact biomass represents a “zero-waste” approach that avoids energy-intensive, chemically harsh downstream extraction processes, aligning this work with green food engineering and realistic industrial feasibility. Furthermore, this approach preserves the holistic nutritional profile of the microalgae and leverages the structural synergy of the intact matrix, where native proteins, cell-wall polysaccharides, and lipids work cooperatively to modulate the starch network. Rather than attempting to isolate individual components, our objective was to comparatively evaluate the physical property changes induced by whole biomass incorporation, establishing a comprehensive baseline template for composite food matrices. A multi-scale analytical approach—combining light microscopy, colorimetry, rheology and near-infrared (NIR) spectroscopy—was used to capture both macro-structural and physical consequences of microalgal addition. Specifically, light microscopy was employed to visualize structural alterations, network integrity and spatial distribution of the biomass and starch granules within the matrix. In addition, colorimetry quantified the impact of microalgal pigments on the optical properties of the final hydrogels. On a macroscopic level, steady rheology was utilized to evaluate changes in flow behavior, apparent viscosity, and shear-thinning characteristics under steady shear conditions, tracking how the biomass alters matrix flow resistance. Complementing these physical assessments, near-infrared (NIR) spectroscopy was leveraged as a non-destructive molecular probe to evaluate alterations in the hydrogen-bonding network and shifts in the water-binding states within the biopolymer system. To our knowledge, this is the first work to assess microalgae-potato starch interactions using an integrated, multi-scale methodology. The findings provide new insights into how microalgal components influence starch network development and support their application not only as nutritional enhancers but also as potential functional modifiers in clean-label hydrocolloid systems and emerging 3D-printed food structures.

## 2. Materials and Methods

### 2.1. Preparation of Samples

Control and fortified starch gels were prepared using potato starch of analytical reagent grade (purity > 99%; containing approximately 20–25% amylose and 75–80% amylopectin) purchased from Panreac Química S.A., Castellar del Vallès, Spain. The commercial microalgal powders utilized were organic *Arthropsira platensis* (Naturitas Essentials, Barcelona, Spain; containing 70.00% protein, 12.00% carbohydrates, 1.00% fat, 0.69% salt, and an estimated moisture content of 5.00–7.00% per total powder weight) and organic *Chlorella vulgaris* (Bibonatur, Madrid, Spain; containing 60.00% protein, 17.00% carbohydrates, 12.00% fat, 11.00% dietary fiber, 0.12% salt, and an estimated moisture content of 5.00–8.00% per total powder weight). Structurally, *Arthrospira platensis* consists of multicellular, open-helix filaments (typically 20–100 μm in length), whereas *Chlorella vulgaris* is characterized by unicellular, micro-spherical cells (typically 2–10 μm in diameter).

The control samples consisted of a 10.00% (*w*/*w*) starch suspension, formulated by dispersing 3 g of potato starch in distilled water to reach a final weight of 30 g. For the fortified formulations, the starch was blended with the respective microalgal biomass at concentrations of 0.500%, 1.00% and 2.00% (*w*/*w*, total formulation basis) while maintaining the potato starch content strictly at 10.00% (*w*/*w*). All samples were executed in triplicate (n = 3) using a completely randomized experimental design.

The preparation followed a standardized thermomechanical procedure to ensure homogeneity and consistent gelation. Initially, the starch and microalgae powders were dispersed in distilled water using a magnetic stirrer at 200 rpm for 10 min, followed by magnetic stirring to ensure homogenous dispersion, and a subsequent hydration period of 30 min at ambient temperature prior to thermal gelatinization. The heating profile was precisely controlled to manage the gelatinization kinetics: the suspensions were heated at a rate of 7 °C/min until reaching 50 °C, then at 5 °C/min to 65 °C, and finally at a slower rate of 1 °C/min until the target temperature of 75 °C was achieved.

Continuous stirring at 200 rpm was maintained throughout the heating phase. Once the system reached 75 °C, the temperature was held constant for 20 min to ensure complete starch granule swelling and amylose leaching. Following the thermal treatment, the gels were cooled to room temperature and subsequently stored at 4 °C overnight to allow for retrogradation and network stabilization. All subsequent physical and chemical analyses were conducted after the samples reached equilibrium at room temperature.

### 2.2. Microscopic Observation

A thin layer of each sample was spread on a slide and stained with Lugol’s iodine solution (analytical grade, containing 5% elemental iodine and 10% potassium iodide) obtained from Panreac Química S.A. (Barcelona, Spain). Following the dye application, the stained samples were allowed to equilibrate for 1 min and were observed immediately under a Nikon Alphashot-2 YS2 compound light microscope at 100× total magnification (Nikon Corp., Tokyo, Japan) [15]. To ensure that the described morphological features were uniform and representative, a minimum of five distinct fields of view were thoroughly scanned per slide across independent batches before capturing representative micrographs. This allows the characterization of the effect of each additive on the starch granule size, structure and arrangement.

Quantitative microstructural characterization of control and microalgae-fortified starch hydrogels (*Arthrospira platensis* and *Chlorella vulgaris* at 0.5%, 1% and 2% *w*/*w*) was conducted using ImageJ software (v.1.53, NIH, Bethesda, MD, USA). Brightfield micrographs were converted to 8-bit gray scale, subjected to rolling-ball background subtraction (radius = 50 pixels), and their threshold was adjusted using standard Otsu binarization to segment the continuous starch matrix and interconnected pore network. Binarized channels were processed using watershed segmentation to resolve adjacent structural domains. Quantitative matrix area fraction (%) and void fraction (%) were extracted directly via phase area measurement. Domain size distribution was determined by measuring individual particle domain areas (*N* = 51–1613 per treatment field), excluding edge-intersecting particles, to establish mean domain area, percentiles (*D*_10_, *D*_50_ and *D*_90_), and domain size span (*Span* *=* (*D*_90_ − *D*_10_)/(*D*_50_)). To evaluate spatial structural homogeneity, a systematic grid array (12 equal sub-regions per frame) was analyzed to record local matrix area fractions (μ), standard deviation (σ), Coefficient of Variation (CV = σ/μ), and Uniformity Index (UI = 1 − CV).

### 2.3. Colorimetric Analysis

The color characteristics of the starch gels were assessed using a MINOLTA tristimulus colorimeter CR-400 (Konica Minolta, Inc., Tokyo, Japan) calibrated against a white ceramic standard. The instrument provided fundamental color coordinates including luminosity (L*)*,* the red-green axis (a*), and the yellow-blue axis (b*). Based on these values, the secondary attributes Chroma (C) (saturation and color intensity) and hue angle (H) (matrix color) were calculated using the following equations:

C = (a*^2^+ b*^2^)^1/2^

H = [arctan(b*/a*) × (180/π)] + k

where k = 360° if arctan(b*/a*) < 0; otherwise, k = 0 (H values were expressed in degrees).

All color data were expressed as an average of 10 independent measurements.

### 2.4. Near-Infrared (NIR) Spectroscopy

Near-infrared (NIR) spectra of the gels were acquired using an Antaris II FT-NIR Analyzer (Thermofisher Scientific, Waltham, MA, USA), fitted with a sample spinner for solid materials. After calibration, absorbance spectra were recorded in the range of 4000–10,000 cm^−1^. All measurements were performed as replicates of three independent batches to ensure spectral reproducibility. Moreover, to guarantee absolute precision during NIR measurements, samples were kept in tightly closed, moisture-resistant containers and scanned rapidly to prevent any ambient moisture evaporation artifacts. To eliminate baseline variations, suppress background noise, and resolve overlapping absorption sub-bands, raw absorbance spectra were transformed into second derivatives using the Savitzky–Golay algorithm. Spectral preprocessing was performed with a frame size of 101 data points and a 2nd-order polynomial fit.

### 2.5. Steady Rheological Measurements and Flow Curve Modeling

A rotational rheometer (HAAKE Rheostress1, Thermoelectron GmbH, Karlsruhe, Germany) equipped with commercial computer software (RheoWin3 Job Manager, version 3.61, Karlsruhe, Germany) was used to measure the rheological properties of the samples.

A concentric rotating cylinder SV2 was used to study samples’ flow under isothermal conditions at 25.0 ± 0.1 °C. Steady-state flow characteristics, apparent yield stress behavior, and thixotropic response were evaluated using a multi-phase shear protocol consisting of four sequential steps. First, no rotation was performed, and the temperature was increased to be stabilized at 25 °C for 240 s. Second, the shear rate was increased logarithmically from 1 to 100 s^−1^ for 90 s. Then, the shear rate was maintained at this value for 60 s. After that, it was again decreased logarithmically to 1 s^−1^ over 90 s. Therefore, three parameters were recorded for each sample: viscosity (η), shear stress (τ) and yield stress.

Experimental shear stress versus shear rate data from the upward ramp were fitted to the Power Law (Ostwald–de Waele) model via non-linear least-squares regression using MATLAB (version R2024a, MathWorks Inc., Natick, MA, USA) to determine the consistency index (K) and flow behavior index (n). From the same upward sweep, the apparent yield stress (τ0) was estimated graphically using the maximum viscosity method by identifying the stress corresponding to the peak apparent viscosity (ƞ_max_) since it is a fast practical approach for biomass-fortified formulations. Finally, thixotropy was determined by calculating the hysteresis loop area enclosed between the upward and downward flow curves.

It is important to note, however, that due to instrumental configuration boundaries, non-destructive oscillatory rheological testing (such as storage and loss moduli, G′ and G″) was outside the scope of this setup.

### 2.6. Statistical Analysis

Data was analyzed using Two-Way Analysis of Variance (ANOVA) followed by Tukey’s Honest Significant Difference (HSD) post hoc test to identify significant differences (*p* < 0.05), with exact significance levels reported down to *p* < 0.001. All analyses were performed in triplicate using IBM SPSS Statistics (Version 30.0), with results presented as mean ± standard deviation. Different superscript letters in the tables indicate statistically significant variations between samples.

## 3. Results and Discussion

### 3.1. Microstructural Analysis and Quantitative Image Profiling

**Figure 1:**
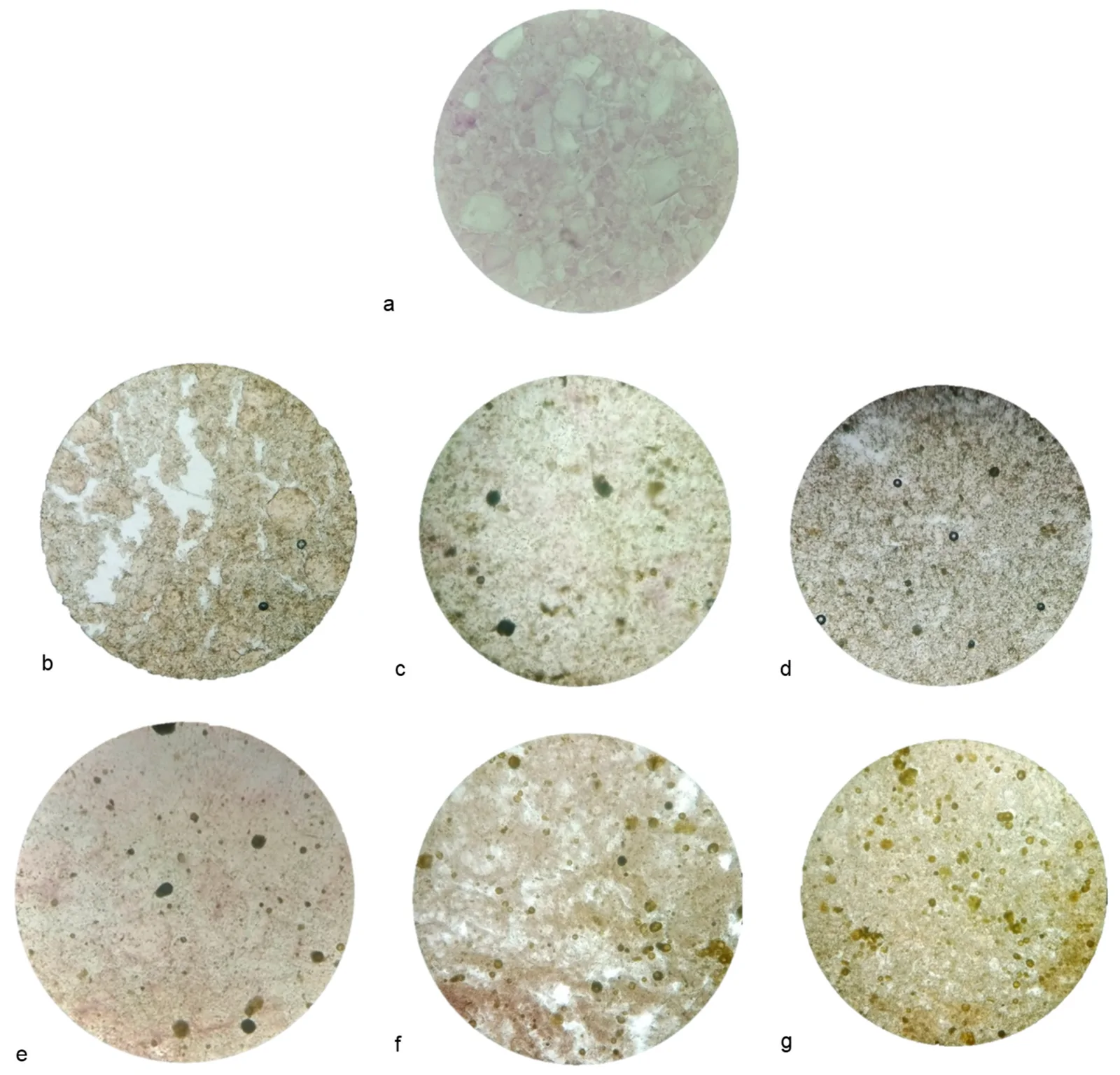
Micrographs of potato starch hydrogels fortified with microalgae at 100× total magnification (10× objective lens; Nikon Alphashot-2 YS2). The full horizontal field-of-view width for each panel represents 1.8 mm. (**a**) Starch gel control. (**b**) *Arthrospira platensis:* 0.5%. (**c**) *Arthrospira platensis:* 1.5%. (**d**) *Arthrospira platensis:* 2%. (**e**) *Chlorella vulgaris:* 0.5%. (**f**) *Chlorella vulgaris:* 1%. (**g**) *Chlorella vulgaris:* 2%.

The microstructural integrity of the potato starch hydrogels was examined following Lugol’s iodine staining (Figure 1), with structural modifications quantitatively characterized via image analysis (Table 1). The control gel exhibited a characteristic honeycomb-like architecture. This structure is characterized by highly swollen, polyhedral starch structures that are densely packed. The dark blue color hues confirm the presence of an amylose-rich continuous phase formed by the leaching of amylose from the granules during hydrothermal treatment. This observation aligns with the foundational principles of starch gelatinization where a cohesive three-dimensional network is established through hydrogen bonding between leached amylose chains [7]. Quantitatively, the control matrix displayed a dominant continuous matrix function (63.65%) and a porosity of 36.35%, with a median domain area (*D*_50_) of 65.00 px^2^ and a Structural Uniformity Index (UI) of 0.90 (CV = 0.10). Overall, the microstructural alterations described below represent an interplay between cellular geometry, cell-wall structural integrity, and the physical presence of leached soluble biomolecules within the continuous starch phase.

#### 3.1.1. Effect of *Arthrospira platensis* (Spirulina) Inclusion

The incorporation of *Arthrospira platensis* biomass induced a concentration-dependent disruption of the starch network (Figure 1b–d). At 0.5% (*w*/*w*), the microstructural network exhibited spatial reorganization, marked by the presence of larger continuous void spaces, which increased the overall void fraction to 44.12% and lowered spatial uniformity (UI = 0.76, CV = 0.24). This structural shift appears to reflect potential physical interference within the amylose matrix by the filamentous biomass fragments, combined with localized hydration competition from leached soluble proteins.

At 1.0% *Arthrospira* platensis, a distinct structural transition occurred: the matrix fraction reached a maximum of 77.71%, accompanied by domain coalescence that increased the median domain area (*D*_50_) to 331.00 px^2^ and the *D*_90_ value to 1290.80 px^2^. This shift indicates localized matrix compaction and domain clustering at this intermediate level. However, at 2.0% inclusion, domain multiplication was observed (N = 1593), returning the median domain size (*D*_50_ = 69.00 px^2^) and porosity (40.59%) closer to baseline levels while achieving high spatial dispersion uniformity (UI = 0.96, CV = 0.04). The visual presence of dense micro-aggregates of starch granules at 2.0% suggests that high biomass concentration may limit full granule expansion, consistent with competitive hydration mechanisms and physical hindrance reported in microalgae–starch composite systems [13].

#### 3.1.2. Effect of *Chlorella vulgaris* Inclusion

Hydrogels fortified with *Chlorella vulgaris* displayed a distinct pattern characterized by discrete, speckled inclusion distribution within the starch network. At 0.5%, *Chlorella vulgaris*, small spherical cells (2–10 μm) were embedded as individual fillers throughout the continuous matrix, maintaining a matrix area fraction of 75.60% and a structural Uniformity Index (UI = 0.90, CV = 0.10) comparable to the control. The small physical size and rigid cellulosic cell wall of *Chlorella vulgaris* cells appeared to permit their integration into the inter-granular regions without causing large-scale void formation.

Increasing *Chlorella vulgaris* fortification to 1.0% led to a localized increase in structural heterogeneity (UI = 0.73, CV = 0.27), with the matrix area fraction decreasing to 59.40% and median domain size reaching 88.00 px^2^ (*D*_90_ = 461.60 px^2^). At 2.0% inclusion, the composite gel reached particulate saturation, characterized by high domain multiplication (N = 1613) and a compact median domain area (*D*_50_ = 65.00 px^2^). The spatial distribution recovered moderate uniformity (UI = 0.85, CV = 0.15), with intact microalgae clusters hypothesized to act as non-interacting physical fillers within the gelatinized matrix. By the 2% *Chlorella* level, the gel reached a state of particulate saturation. The microstructure was characterized by dense clusters of microalgae cells that acted as fillers within the starch matrix. A comparative study on rice starch gels similarly found that *Chlorella* tended to promote a more homogeneous composite structure than larger microalgae species, though it significantly increased the gelatinization temperature due to the high cellulose content of its cell walls [13]. Moreover, in all microalgae-fortified samples, microalgal biomass appeared as intact pigmented cells spread within the matrix.

### 3.2. Impact of Microalgal Fortification on Potato Starch Gel Color

Color properties of the potato starch hydrogels were significantly affected by microalgae species, biomass concentration and their interaction term across all CIELAB coordinates (*p* < 0.05; Table 2). The extreme significance observed (*p* < 0.001 for most factors) reflects the high chromophoric potency of microalgal pigments- primarily phycocyanin in *Arthrospira platensis* and chlorophyll a/b in *Chlorella vulgaris*- combined with high measurement precision (N = 10).

The unfortified control hydrogel displayed a baseline lightness (L*) of 31.59 ± 1.79 and a positive red/magneta coordinate (a* = 6.09 ± 0.54). Because native gelatinized starch is semi-translucent, incident light penetrates the matrix and is partially absorbed by the underlying measurement stage during reflectance testing, yielding a lower lightness baseline than completely opaque matrices. Furthermore, the slightly positive baseline a* value is attributed to minor thermal browning products and trace native potato phenolic interactions generated during high-temperature starch gelatinization during high temperature starch gelatinization (90 °C).

Incorporation of microalgal biomass induced a significant reduction in lightness (L*, *p* < 0.001), driven by intense light absorption by cellular pigments. A strong species-dependent darkening effect was recorded for *Arthrospira platensis*, reaching a minimum lightness of 18.54 ± 2.11 at 2.0% inclusion. In contrast, hydrogels fortified with *Chlorella vulgaris* maintained a stable lightness level (24.41–26.01) across all concentrations, demonstrating that *Chlorella vulgaris* cells preserve light reflection more effectively within the gel network than the dark phycobiliprotein matrix of *Arthrospira platensis*.

Chromaticity parameters (a*, b* and C*) demonstrated highly significant interaction effects between species and concentration (*p* < 0.001). Addition of microalgae shifted the greenness coordinate (a*) from positive control values into the negative spectrum (down to −2.36 ± 0.05 for 1.0% *Chlorella vulgaris*). *Chlorella vulgaris* fortification imparted a pronounced yellow-green hue, marked by a sharp increase in b* (up to 8.49 ± 0.25) and total chroma (C* = 8.81 ± 0.23), possibly reflecting the high concentration of lutein and chlorophylls. Conversely, *Arthrospira platensis* hydrogels exhibited markedly lower chroma (C* = 1.52–2.00), producing dark, desaturated olive-to-blush tones.

The hue angle (H) further illustrated the distinct chromatic trajectories of the two species (*p* < 0.05). The control gel exhibited a baseline hue angle of 322.09 ± 4.55°, situated in the red-purple quadrant. Fortification with *Chlorella vulgaris* shifted the hue angle into the 285.70–298.08° region, characteristic of a stable green-yellow chromatic response. For *Arthrospira platensis*, hue values resided near the 0°/360° quadrant boundary (32.30° at 0.5% and 346.28° at 1.0%), reflecting a dark, blue-green tone where a* fluctuates near zero.

Factorial analysis demonstrates that color expression is governed by species-specific pigment structures interacting within the gelatinized starch network. These distinct colorimetric footprints offer practical tailoring options for functional food design. *Chlorella vulgaris* provides warm, vibrant green-yellow pigmentation suitable for spreadable matrices, while *Arthrospira platensis* delivers deep, low-chroma tones desirable for dark, rich functional snack formulations.

### 3.3. Molecular Interactions and Water State Analysis via NIR Spectroscopy

**Figure 2:**
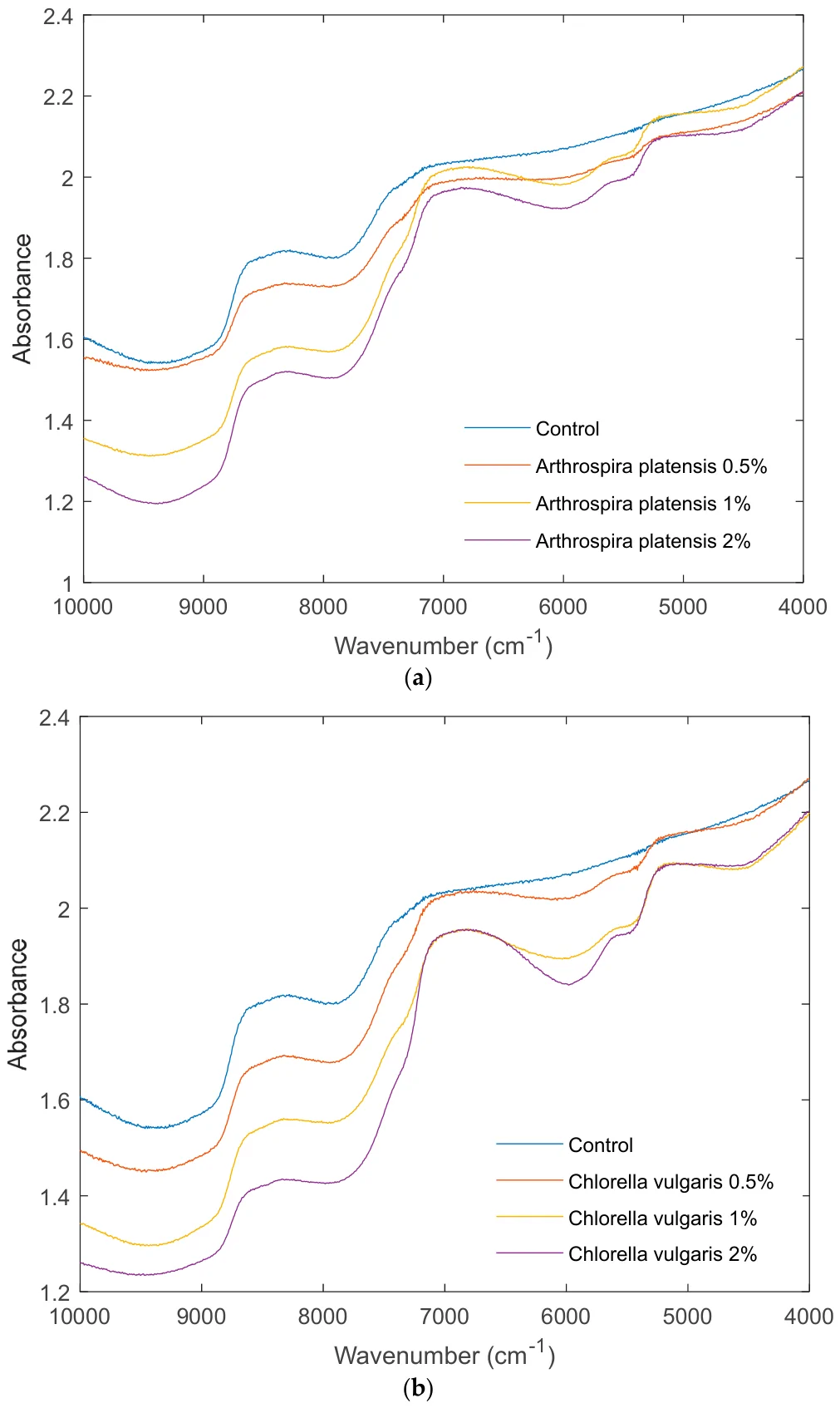

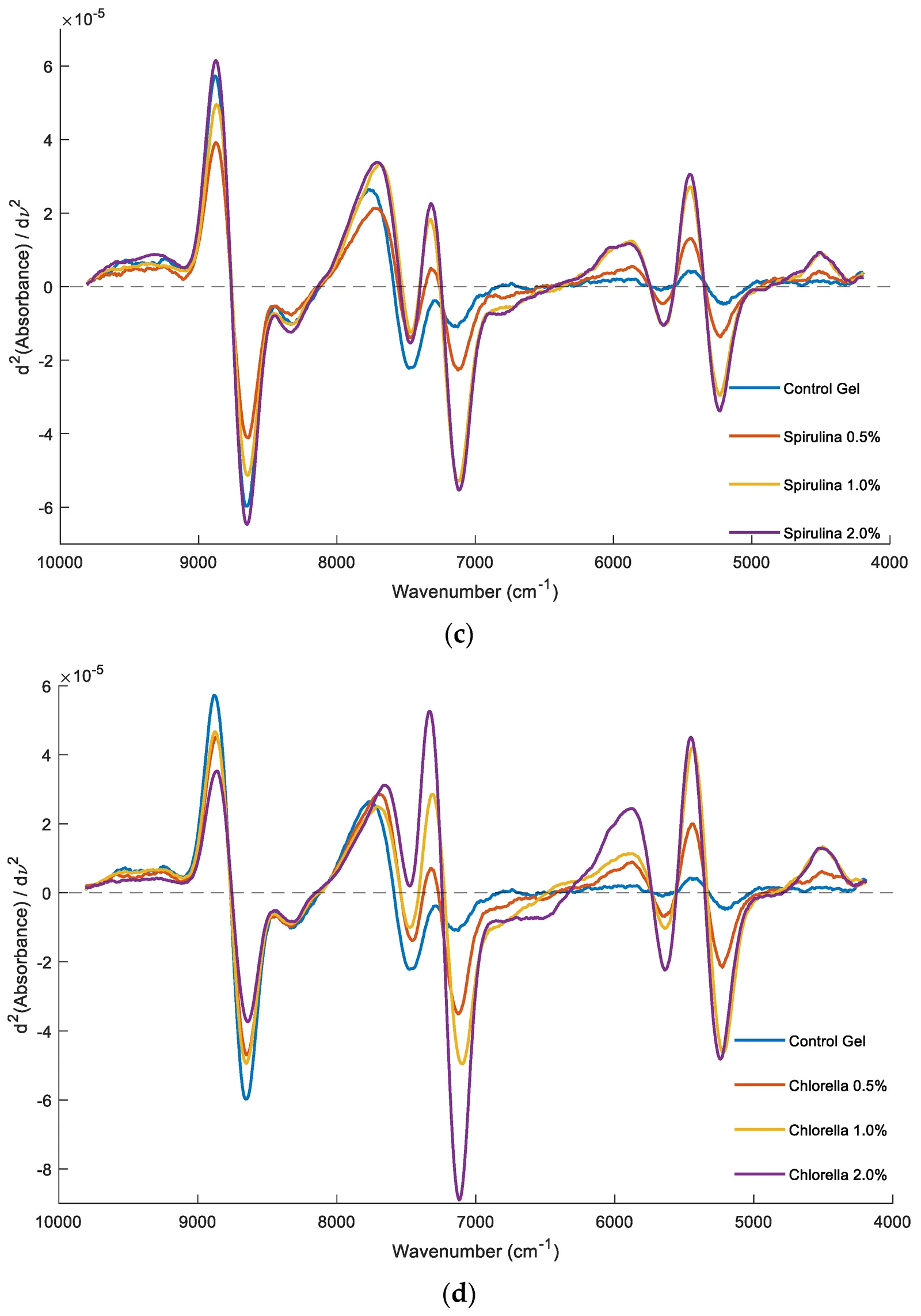
Near-infrared (NIR) spectra of potato starch hydrogels fortified with *Arthrospira platensis* (*Spirulina*) and *Chlorella vulgaris* at different concentration (0%, 0.5%, 1% and 2%): (**a**) raw NIR spectra (4000–10,000 cm^−1^) of *Spirulina*-fortified hydrogels; (**b**) raw NIR spectra (4000–10,000 cm^−1^) of *Chlorella vulgaris* fortified hydrogels; (**c**) second-derivative spectra (Savitzky–Golay filter, 101-point frame size, 2nd-order polynomial) of *Spirulina*-fortified hydrogels; and (**d**) second-derivative spectra of *Chlorella vulgaris*-fortified hydrogels.

Near-infrared (NIR) spectra of the control and microalgae-fortified potato starch hydrogels were evaluated across the complete 4000–10,000 cm^−1^ spectral region (Figure 2a,b). To eliminate baseline variations and resolve overlapping absorption sub-bands, second-derivative transformations were calculated using a Savitzky–Golay filter (101-point window size, second-order polynomial) (Figure 2c,d).

In the raw NIR spectra, the primary absorption features across all hydrogel formulations are dominated by two broad, intense regions: 6800–7000 cm^−1^ and 5100–5200 cm^−1^ (Figure 2a,b). These bands correspond to the first overtone of O-H stretching vibrations (~6900 cm^−1^) and the primary combination band (O-H stretch + H-O-H bending, ~5172 cm^−1^) of matrix water and starch hydroxyl groups [16]. In the control hydrogel, these features reflect a fully gelatinized, uniform polysaccharide matrix wherein water molecules predominantly reside in a bulk, hydrogen-bonded state within the swollen starch network.

Upon the incorporation of *Arthrospira platensis* and *Chlorella vulgaris* (0.5–2.0% *w*/*w*), dose-dependent variations in band intensity and shape are observed. Rather than indicating chemical reaction or new covalent complexation, these spectral shifts suggest localized water redistribution within the composite gel matrix. The presence of insoluble microalgal biomolecules (proteins, structural lipids, and cell-wall polysaccharides) alters the spatial availability and local hydrogen-bonding environment of water surrounding the gelatinized starch polymers, leading to possible attenuation of bulk water absorption features [17].

Because overlapping broad bands in raw spectra can mask subtle chemical signals, second-derivative analysis was performed to pinpoint specific vibrational markers (Figure 2c,d). In the second-derivative profiles, the minimum of the primary water combination bands resolves cleanly at 5172 cm^−1^. The deepening and minor position shifts in this 5172 cm^−1^ derivative valley in fortified samples further reflect alterations in the state of matrix-bound water. Crucially, a distinct downward derivative valley emerged exclusively in the fortified samples at 5646 cm^−1^, exhibiting an intensity that scaled directly with microalgae concentration. This feature is assigned to the first overtone of aliphatic C-H stretching vibrations (2νC-H) characteristic of microalgal membrane lipids and cell-wall biopolymers [17].

The appearance of the 5646 cm^−1^ marker in second-derivative spectra provides non-destructive evidence of the physical integration and uniform dispersion of whole microalgae biomass within the potato starch hydrogel. However, in the absence of quantitative sorption isotherms or water activity (a_w_) measurements, these spectral changes are interpreted strictly as physical entrapment and water phase redistribution rather than the formation of a distinct ternary biopolymer complex or active water competition.

From an analytical perspective, second-derivative NIR spectroscopy demonstrates clear utility as a rapid, non-destructive quality control tool for functional food manufacturing. The progressive deepening of the 5646 cm^−1^ derivative valley enables the qualitative verification of biomass dosage accuracy in microalgae-enriched starch systems. Furthermore, monitoring shifts in the 5172 cm^−1^ water combination region provides a predictive means to predict potential physical stability and moisture reorganization during storage, offering a non-invasive complement to destructive textural characterization.

### 3.4. Steady Rheological Parameters

#### 3.4.1. Viscosity and Flow Curve Modeling

**Figure 3:**
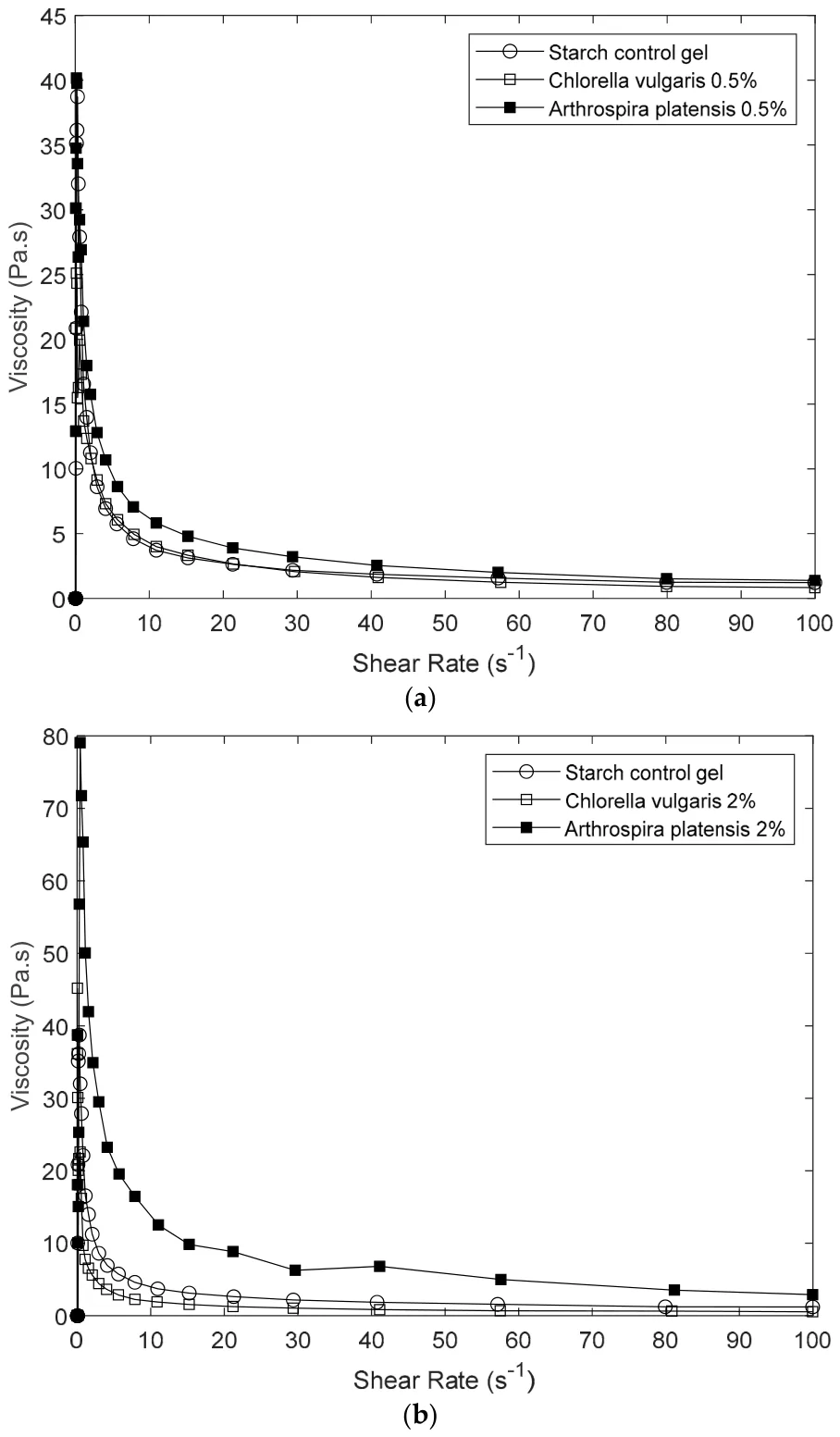
Typical flow curve of potato starch control gel alone and with different additives at 0.5% concentration (**a**) and at 2% concentration (**b**).

The steady-shear rheological curves for the control and fortified potato starch gels reveal a characteristic non-Newtonian, shear-thinning behavior across all samples, as evidenced by the sharp decrease in apparent viscosity with increasing shear rate (Figure 3).

At a low inclusion level of 0.5%, the *Arthrospira platensis* gel exhibited a higher initial viscosity compared to the control, likely due to the reinforcing effect of its long filaments combined with soluble biomolecules that possibly leached into the continuous phase. Conversely, the *Chlorella vulgaris* 0.5% sample tracked more closely with the control, suggesting that its small, spherical cells, characterized by rigid cellulosic cell walls, integrate into the inter-granular spaces without significantly increasing flow resistance (Figure 3a). This divergence became more pronounced at the 2% saturation level; the *Arthrospira platensis* 2% sample reached a peak viscosity of approximately 80 Pa·s, while the *Chlorella vulgaris* 2% sample remained less viscous than the control (Figure 3b). This contrast possibly reflects a multi-faceted interplay between cell shape, cell wall composition, and leached cellular constituents: the filamentous nature of *Spirulina* promotes physical entanglement with leached amylose chains, creating a crowded matrix that obstructs flow, whereas the spherical *Chlorella vulgaris* cells can act as micro-spacers or lubricants that disrupt the continuity of the starch network without providing the same mechanical reinforcement. These macro-scale observations are supported by NIR data, specifically the water-binding peak at 5172 cm^−1^, which indicates that competitive hydration at 2% inclusion leads to a more concentrated and pastier continuous phase in *Spirulina* gels. Ultimately, these results align with recent findings suggesting that while Spirulina is well-suited for formulations requiring high structural stability and shape retention under structural loads, Chlorella is better suited for products where nutritional fortification must be achieved without inducing excessive thickening or reducing flowability [13].

To quantitatively describe these flow profiles, experimental shear stress versus shear rate data were modeled using the Power Law (Ostwald–de Waele) equation (Table 3). The Power Law model provided high goodness-of-fit values (R^2^) across all formulations (0.97–0.99). Across all control and microalgae-fortified hydrogels, the flow behavior index (n) remained strictly below unity (0.36–0.46), mathematically confirming non-Newtonian, shear-thinning (pseudoplastic) behavior. This pseudoplastic nature is a characteristic of gelatinized starch networks, where applied shear forces induce progressive untangling and directional alignment of leached amylose and amylopectin chains along the flow vector. Notably, while biomass incorporation maintained this fundamental pseudoplastic character, it significantly altered the flow resistance of the gel matrix as reflected by changes in the consistency index (K).

The consistency index (K), which reflects apparent viscosity at low shear rates, exhibited distinct species- and concentration-dependent variations. For *Arthrospira platensis* hydrogels, addition of 0.5% and 2% elevated K values (23.92 and 53.32 Pa·s^n^). This marked increase points to possible physical and chemical interactions between *Arthrospira platensis* filamentous cells, cell walls and released cellular molecules with the continuous gelatinized gel matrix, thereby increasing flow resistance. Conversely, *Chlorella vulgaris* incorporation generally yielded lower consistency indices (7.90–16.96 Pa·s^n^). This drop suggests that the rigid, small spherical cells, cellulosic cell walls and cellular-released molecules from *Chlorella vulgaris* act as non-reinforcing fillers or spacers that interrupt the continuous starch matrix and reduce the viscosity. Interestingly, both microalgal species exhibited an intermediate drop in K at 1% inclusion (3.84 and 4.46 Pa·s^n^), suggesting a critical concentration threshold where phase competition or spatial reorganization of the starch network temporarily lowers flow resistance before bulk physical packing dominates.

#### 3.4.2. Yield Stress

**Figure 4:**
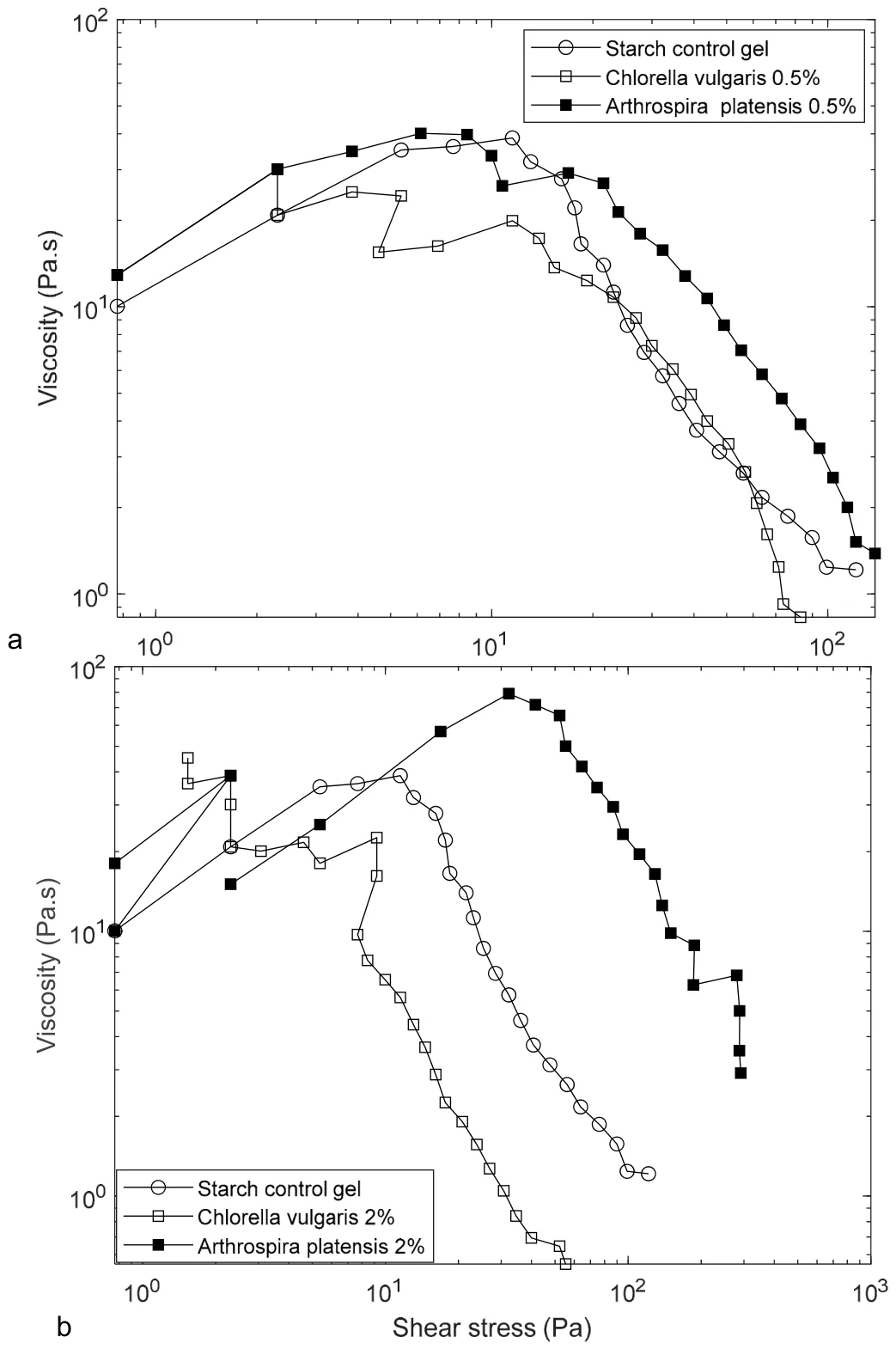
Viscosity versus shear stress in potato starch gel control and fortified samples at (**a**) 0.5% and (**b**) 2% (*w*/*w*) concentrations.

The shear stress-viscosity profiles provide a detailed map of the mechanical threshold at which each gel transitions from a structured solid to a flowing fluid (Figure 4).

At low stress levels, the samples maintain a stable or slightly increasing viscosity, indicating that the internal network of hydrogen bonds and physical entanglements remains intact. However, once the applied stress reaches a critical value-the yield stress-, the viscosity drops precipitously across several orders of magnitude, signifying the abrupt rupture of the biopolymer network. This yield stress value was determined by identifying the point of maximum viscosity (ƞ_max_) just prior to this structural breakdown. The results revealed a species- and concentration-dependent modulation of gel strength, while the starch control exhibited a yield stress of approximately 10.2 Pa, fortification with *Arthrospira platensis* significantly reinforced the matrix, elevating the yield stress to 11.5 Pa at 0.5% inclusion and reaching a substantial 32.4 Pa at the 2% level. This behavior is consistent with the enhanced physical entanglement and competitive moisture binding described in Section 3.4.1.

#### 3.4.3. Thixotropy

**Figure 5:**
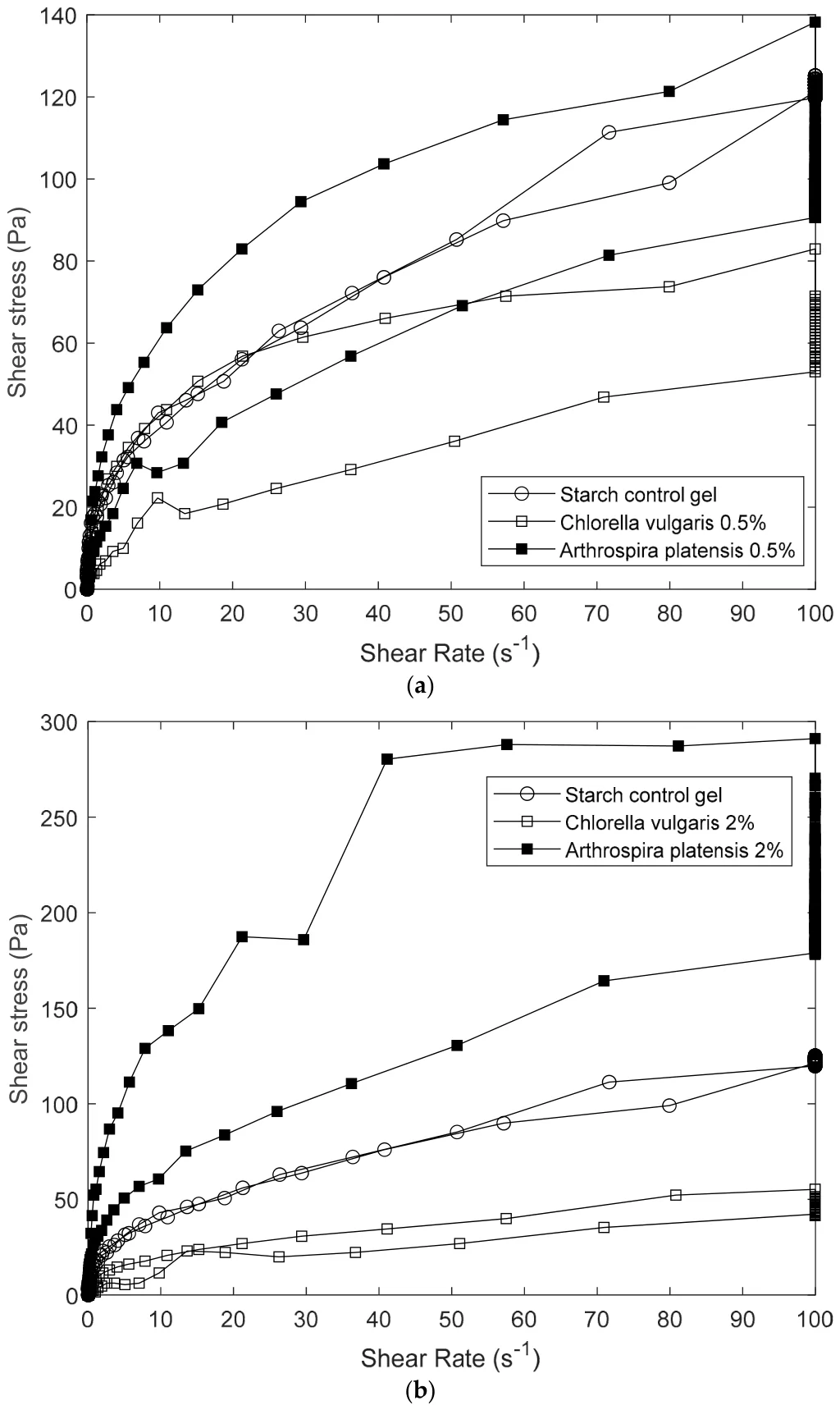
Hysteresis loops in potato starch gel control and fortified samples at (**a**) 0.5% and (**b**) 2% (*w*/*w*) concentrations.

The time-dependent rheological behavior of the potato starch gels was evaluated through the hysteresis loop analysis (Figure 5).

It was created by subjecting the potato purée control and fortified samples to a rising shear rate (from 1 to 100 s^−1^ for 90 s), a constant shear rate for 60 s, and a decreasing shear rate (from 100 to 1 s^−1^ for 90 s). The area between the upward and downward flow curves serves as a direct measure of thixotropy. The starch control gel displayed a rhombus-like hysteresis loop, characterized by an upward curve that follows a higher shear stress path than the downward curve. This shape indicates a time-dependent loss of structural integrity, as the undisturbed amylose–amylose junctions offer maximum resistance to flow initially but are physically ruptured and aligned under increasing shear rate. Because the system cannot instantaneously rebuild these hydrogen-bonded junctions when the shear is reduced, it follows a lower stress path upon the return, creating the visible rhombus area. While the control showed a moderate thixotropic baseline, the response was highly sensitive to microalgae fortification; the *Arthrospira platensis* 2% gel displayed a significantly wider loop, suggesting that its complex, entangled filamentous network undergoes a substantial shear-induced disruption and requires a longer recovery time to reform. Conversely, the *Chlorella vulgaris* gels showed narrower hysteresis areas, particularly at the 2% level, implying a more fragile but rapidly recovering structure where the spherical micro-particles and intact cell-wall structures minimize long-range entanglements. These differences in structural memory are of high industrial significance, as the high thixotropy of *Arthrospira platensis* indicates a shear-thinning and slow thickening profile, whereas the low thixotropy of *Chlorella vulgaris* suggests a more stable consistency during continuous processing.

While a definitive evaluation of the true linear viscoelastic network parameters requires oscillatory rheology (G′ and G″), the significant modifications observed in yield stress and hysteresis loop area demonstrate that whole microalgal biomass introduces species-specific physical flow resistance within the potato starch matrix, providing practical insights for formulating functional food systems.

## 4. Conclusions

This study demonstrated that fortifying potato and starch gels with microalgae significantly modifies their rheological, colorimetric, and molecular properties. Quantitatively *Arthrospira platensis* fortification (0.5–2.0% *w*/*w*) increased yield stress (from 10.20 Pa in the control to 32.4 Pa at 2.0% inclusion level) and elevated high-shear viscosity, which is likely associated with its filamentous nature, cell-wall composition and leached biomolecules. Conversely, spherical *Chlorella vulgaris* cells appeared to act as physical filler particles, reducing yield stress (to 4.80 Pa) and initial viscosity, while expanding the structural hysteresis loop area. Color analysis revealed a marked dose-dependent reduction in lightness (L* decreasing from 31.59 to 18.54 for *Spirulina* 2.0% inclusion level) alongside species-specific chromatic shifts in accordance with the dominant pigment profiles of the used microalgae. In second-derivative NIR spectra, the emergence of the aliphatic C-H overtone at 5646 cm^−1^ and depth modulation of the O-H combination band at 5172 cm^−1^ confirmed physical biomass dispersion and water matrix redistribution.

Directly addressing the primary research objective, this study reveals that whole microalgae can act as effective physical composite modifiers rather than passive nutrient carriers within gelatinized starch hydrogels. These empirical observations provide practical insights into food engineering, enabling the formulation of either high-stability matrices with enhanced structural retention or smooth, low-viscosity functional food systems. However, the scope of this study is limited to the immediate physical, optical, and flow properties of the fresh hydrogels, focusing on a single starch variety and a narrow range of fortification levels. These were conducted under controlled laboratory conditions, which may not fully account for the complexities of multi-ingredient food systems containing lipids or electrolytes. Future research should therefore evaluate classical hydrocolloid properties such as long-term retrogradation kinetics, syneresis, freeze–thaw stability, and formal texture profile analysis (TPA). Additionally, expanding to comprehensive sensory evaluations will help determine how these rheological shifts influence consumer mouthfeel and flavor release. Exploring the synergistic effects of combining microalgae with other hydrocolloids will also be essential. Finally, assessing the metabolic bioavailability of nutrients entrapped within these modified starch networks will help optimize the shelf-life and nutritional impact of next-generation sustainable food products.

Overall, this work provides a comprehensive physical evaluation demonstrating that incorporating distinct microalgal species offers a viable route to modify the rheological, structural, and optical performance of potato starch-based systems.

## Figures and Tables

**Table 1:** A table showing the quantitative analysis of potato starch gel micrographs.

Quantitative Parameters	Starch Gel Control	*Arthrospira platensis*			*Chlorella vulgaris*		
		0.5%	1%	2%	0.5%	1%	2%
Phase Fraction Analysis							
Matrix Area %	63.65	55.88	77.71	59.41	75.60	59.40	60.67
Porosity %	36.35	44.12	22.29	40.59	24.40	40.60	39.33
Particle Distribution Analysis							
Total Domain Count (N)	51.00	1305.00	453.00	1593.00	1088.00	973.00	1613.00
Mean Domain Area (px^2^)	110.25	141.87	545.32	108.59	216.31	183.71	105.98
*D*_10_ (px^2^)	25.00	25.00	57.00	27.00	34.00	25.00	26.00
*D*_50_ (Median Domain Area, px^2^)	65.00	63.00	331.00	69.00	121.00	88.00	65.00
*D*_90_ (px^2^)	240.00	257.60	1290.80	236.00	479.30	461.60	223.60
Domain Size Span ((*D*_90_ − *D*_10_)/*D*_50_)	3.31	3.69	3.73	3.02	3.68	4.96	3.04
Spatial Structural Uniformity							
Grid Mean Area (μ, %)	61.68	53.71	78.14	59.13	75.10	56.79	60.13
Grid Standard Deviation (σ)	5.88	12.98	6.76	2.53	7.85	15.14	9.22
Coefficient of Variation (CV = σ/μ)	0.10	0.24	0.09	0.04	0.10	0.27	0.15
Uniformity Index (UI = 1 – CV)	0.90	0.76	0.91	0.96	0.90	0.73	0.85

**Table 2:** Effect of *Arthrospira platensis* and *Chlorella vulgaris* concentration on the colorimetric parameters (L*, a*, b*, C* and h°) of potato starch hydrogels. Values are expressed as mean ± standard deviation (n = 10). Different superscript letters within the same column indicate statistically significant differences (*p* < 0.05) according to Tukey’s HSD and Two-Way ANOVA tests.

Starch Gel Sample	Additive Concentration (% *w*/*w*)	L*	a*	b*	C* (Chroma)	h° (Hue)
**Control**	-	31.59 ± 1.79 ^c^	6.09 ± 0.54 ^d^	−4.75 ± 0.63 ^a^	7.74 ± 0.54 ^d^	322.09 ± 4.55 ^a,b^
***Arthrospira platensis***	0.5	25.65 ± 0.99 ^b^	−1.46 ± 0.18 ^b^	−1.03 ± 0.62 ^b^	1.86 ± 0.37 ^a^	32.30 ± 18.70 ^c^
	1	24.57 ± 1.45 ^b^	−1.75 ± 0.24 ^b^	0.48 ± 0.09 ^c^	2.00 ± 0.28 ^a,b^	346.28 ± 25.67 ^b^
	2	18.54 ± 2.11 ^a^	0.40 ± 0.06 ^c^	1.25 ±0.06 ^c^	1.52 ± 0.03 ^a^	29.91 ± 2.83 ^c^
***Chlorella vulgaris***	0.5	26.01 ± 1.12 ^b^	−1.53 ± 0.03 ^b^	2.91 ± 0.61 ^d^	3.30 ± 0.06 ^b,c^	298.08 ± 4.19 ^a^
	1	24.41 ± 3.40 ^b^	−2.36 ± 0.05 ^a^	8.49 ± 0.25 ^e^	8.81 ± 0.23 ^d^	285.70 ± 1.39 ^a^
	2	25.93 ± 1.74 ^b^	−1.39 ± 0.05 ^b^	4.14 ± 0.30 ^d^	4.38 ± 0.04 ^c^	288.15 ± 5.20 ^a^
**Two-Way** **ANOVA (** ***p*** ** values)**						
**Species**		<0.001	<0.001	<0.001	<0.001	<0.001
**Concentration**		<0.001	<0.001	<0.001	<0.001	<0.05
Interaction (Species* Concentration)		<0.001	<0.001	<0.001	<0.001	<0.05

**Table 3:** Power Law model parameters (K, n) and goodness-of-fit (R^2^) for control and microalgae-fortified potato starch hydrogels.

Sample	Additive Concentration (%)	Consistency Index (K) (Pa·s^n^)	Flow Behavior Index (n)	Goodness of Fit (R^2^)
**Starch gel control**	0	15.18	0.44	0.99
***Arthrospira platensis*** ** fortified starch gel**	0.5	23.92	0.39	0.99
	1	3.84	0.46	0.99
	2	53.32	0.39	0.97
***Chlorella vulgaris*** ** fortified starch gel**	0.5	16.96	0.36	0.97
	1	4.46	0.39	0.99
	2	7.90	0.42	0.99

## Data Availability

The original contributions presented in this study are included in the article. Further inquiries can be directed to the corresponding authors.
